# Towards a Transferable Modeling Method of the Knee to Distinguish Between Future Healthy Joints from Osteoarthritic Joints: Data from the Osteoarthritis Initiative

**DOI:** 10.1007/s10439-023-03252-8

**Published:** 2023-06-07

**Authors:** Alexander Paz, José J. García, Rami K. Korhonen, Mika E. Mononen

**Affiliations:** 1 Department of Technical Physics, University of Eastern Finland, Yliopistonranta 1, 70211 Kuopio, Finland; 2 Escuela de Ingeniería Civil y Geomática, Universidad del Valle, Cali, Colombia

**Keywords:** Knee, Cartilage, Osteoarthritis, Finite element modeling, Transferability

## Abstract

Computational models can be used to predict the onset and progression of knee osteoarthritis. Ensuring the transferability of these approaches among computational frameworks is urgent for their reliability. In this work, we assessed the transferability of a template-based modeling strategy, based on the finite element (FE) method, by implementing it on two different FE softwares and comparing their results and conclusions. For that, we simulated the knee joint cartilage biomechanics of 154 knees using healthy baseline conditions and predicted the degeneration that occurred after 8 years of follow-up. For comparisons, we grouped the knees using their Kellgren–Lawrence grade at the 8-year follow-up time and the simulated volume of cartilage tissue that exceeded age-dependent thresholds of maximum principal stress. We considered the medial compartment of the knee in the FE models and used ABAQUS and FEBio FE softwares for simulations. The two FE softwares detected different volumes of overstressed tissue in corresponding knee samples (*p* < 0.01). However, both programs correctly distinguished between the joints that remained healthy and those that developed severe osteoarthritis after the follow-up (AUC = 0.73). These results indicate that different software implementations of a template-based modeling method similarly classify future knee osteoarthritis grades, motivating further evaluations using simpler cartilage constitutive models and additional studies on the reproducibility of these modeling strategies.

**Supplementary Information:**

The online version contains supplementary material available at 10.1007/s10439-023-03252-8.

## Introduction

Articular cartilage is a fibril-reinforced biphasic tissue that offers frictionless contact and impact absorption functions in the knee joint for many years of an individual’s life. Unfortunately, a variety of risk factors such as aging, joint injury, and being overweight [3] may impair cartilage optimal functioning, leading to a degenerative condition called osteoarthritis (OA). Therefore, understanding OA driving mechanisms is key to developing treatment strategies for this painful condition [11].

Currently, there is no clinical method for personalized risk estimations for the onset and progression of knee OA. In this regard, computer-based approaches have been recently utilized to simulate the biomechanics of the knee in healthy and diseased conditions [27]. The results of those simulations, e.g., stress and strain distributions, can be further utilized to optimize surgical procedures or preventive actions [12]. For instance, by identifying loadings that cause abnormal cartilage hip mechanics [13] or minimizing bone stress concentrations after ankle fracture reductions [18]. However, regarding conservative measures, such as weight loss or gait retraining, validated computational approaches are missing to evaluate the personalized effects of those measures with simulated disease progression.

Modeling the biomechanics of living tissues in the human body is challenging due to the complex geometries and loads they bear. For the knee joint, several strategies based on finite element analysis (FEA) have been developed, accounting for the complex tissue structure, subject-specific geometries, and loading conditions of the joint [2, 8, 24, 30]. In addition, when the studies involve cohort data, generating and running a large number (*N* > 100) of personalized models are time-consuming processes [32]. In this regard, Mononen et al. (2019) proposed a template-based approach to rapidly simulate the biomechanics of the medial compartment of the knee using FEA. Yet, they modeled only 21 subjects, which could represent a limitation for the reliability of the approach. Moreover, they used a user-defined cartilage formulation in ABAQUS, which is a commercial software not readily available to many users, possibly limiting the use of the method by researchers worldwide. Approaches like this should be replicated with a large patient population and by using more accessible tools, including freely available FE software like FEBio [25], which is receiving increasing attention to model multiphasic problems in biomechanics due to its flexibility given by a large library of non-linear constitutive equations that are easily implementable. More accessible methods would enable faster development of novel strategies that could help in clinical decision-making on subject-specific conservative measures. For instance, by visualizing abnormally loaded regions in the joint and simulating preventive interventions to alleviate those harmful conditions, as well as by offering means to quantify the risk for OA.

In this study, we extended the template-based method [26] to over 100 joints to evaluate its capability for predicting the development of knee osteoarthritis. We generated the models and reproduced the results by ABAQUS and FEBio finite element softwares by using baseline and follow-up information from subjects from the Osteoarthritis Initiative (https://nda.nih.gov/oai/). We hypothesize that the template-based FEA pipeline, implemented in two different finite element softwares, distinguishes subjects at high risk for knee OA from those at low risk, even though all subjects have similar demographic characteristics and healthy radiographic knee conditions at baseline.

## Materials and Methods

**Fig. 1:**
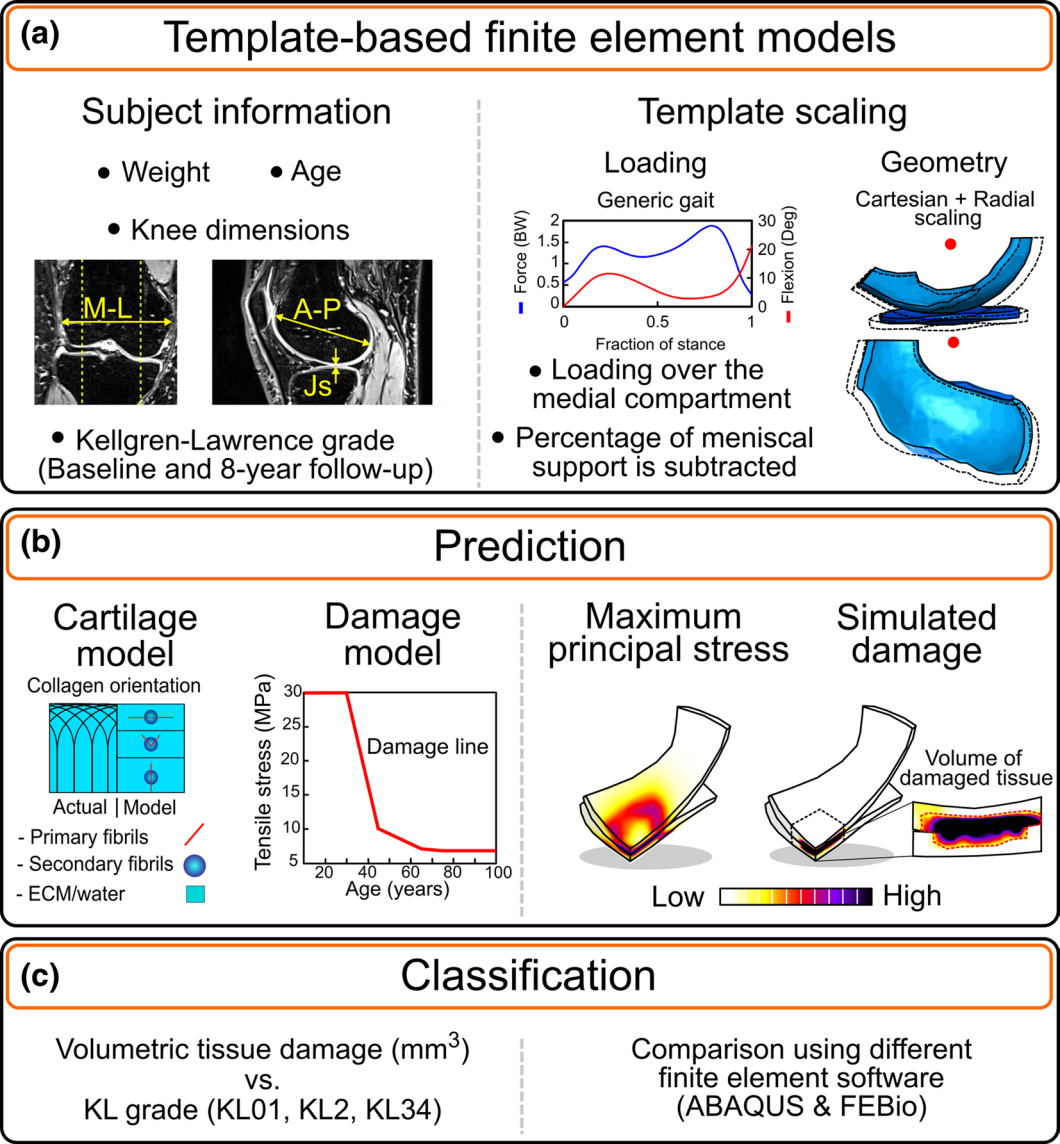
Workflow of the present study. **a** Generation and simulation of the template-based finite element models. **b** Biphasic and fibril-reinforced models of articular cartilage as well as a method to define the volume of tissue at risk for damage. **c** Evaluation of the subject classification based on volume at risk for the various KL grades and a comparison between the two most commonly used FEA software (ABAQUS vs FEBio)

Figure 1 overviews the workflow of this study. First, medial knee compartment FE models were generated using the template-based approach to simulate the stance phase of the gait [26]. For that, we used anatomical measurements from the distal femur and tibiofemoral joint space obtained from clinical MRI, and demographic information (age and weight) of the subjects at a common healthy baseline. Then, we used age-dependent thresholds of maximum principal stresses, based on reported experimental observations of monotonic tensile tests of human cartilage samples [17, 26], to compute the volume of degenerated cartilage, assuming that the degeneration initiates in the collagen network [14, 21]. Finally, we compared degenerated volumes between healthy and OA knees, classified based on radiographic evidence at the 8th year of follow-up, using two different finite element (FE) softwares: FEBio [25] (FEBio version 3.3.2) and ABAQUS (v.2018, Dassault Systèmes Simulia Corp., Providence, RI).

### Template-Based Finite Element Models

#### Subjects Sample

**Fig. 2:**
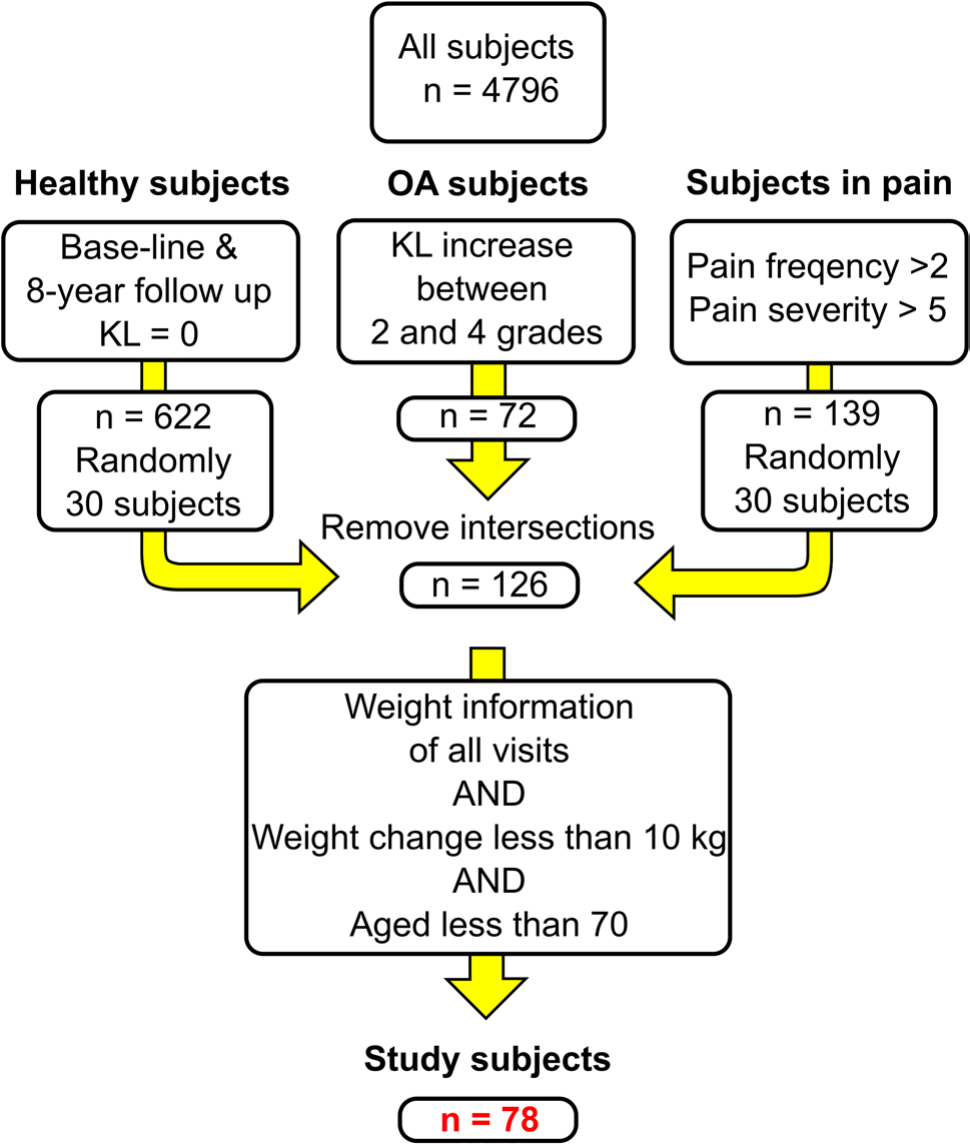
Inclusion criteria to select the subjects from the OAI database. Pain frequency > 2 indicates daily to constant knee pain, and pain severity > 5 refers to scores in the upper half of a self-reported pain level from 0 (“no pain”) to 10 (“pain as bad as you can imagine”). The final number of subjects was 78 (154 knee models). Namely, 88 knees with KL grade 0, 15 with KL1, 36 with KL2, and 15 accounting for knees with KL3 and KL4

We obtained baseline and 8-year follow-up information from 78 subjects from the Osteoarthritis Initiative database. This number of subjects corresponds to 154 knees since the information from both knees was not available for all the subjects. Figure 2 shows the inclusion criteria for the subject selection. The data used in the study included MRI images and Kellgren–Lawrence (KL) scores for the left and right knees, as well as the subjects' weight and age.

Anatomical dimensions of the distal femur and tibiofemoral joint space were measured for all the knees at the healthy baseline (KL grade 0 or 1) and used to scale a template finite element model (Fig. 1a) [26]. The dimensions correspond to the medial–lateral maximum condylar distance (M–L), the maximum anterior–posterior distance (A–P) measured in the sagittal plane of the medial condyle, and the joint space (JS) measured in the same plane as the A–P parameter. The knees were grouped based on their KL status at the 8-year follow-up. We will refer to individuals with KL = 0 (KL0) and KL = 1 (KL1) as healthy, KL = 2 (KL2) as mildly affected, and KL = 3 and KL = 4 (KL34) as severely affected by OA. Table 1 summarizes the subject characteristics and measurements (a visual representation of the information can be found in Supplementary Material, Section 1).

**Table 1:** Baseline characteristics of the 154 knees (mean ± standard deviation) used in this study

	KL0	KL1	KL2	KL34	All
*N* (left/right)	88 (46/42)	15 (6/9)	36 (15/21)	15 (10/5)	154 (77/77)
Age (years)	56.6 ± 5.6	51.6 ± 4.4	58.0 ± 5.5	57.6 ± 7.4	56.6 ± 5.9
Weight (kg)	76.1 ± 16.4	77.6 ± 17.8	74.8 ± 14.4	84.6 ± 15.0	76.8 ± 16.0
BMI (kg m^−2^)	27.2 ± 4.2	28.8 ± 6.9	28.0 ± 5.2	30.1 ± 5.2	27.8 ± 5.1
M-L (mm)	80.4 ± 6.1	77.4 ± 6.5	77.8 ± 6.3	78.8 ± 6.4	79.4 ± 6.3
A-P (mm)	55.0 ± 4.8	52.6 ± 3.6	54.0 ± 3.7	54.8 ± 4.7	54.5 ± 4.4
JS (mm)	5.0 ± 0.8	4.8 ± 0.6	4.9 ± 0.7	5.0 ± 0.9	4.9 ± 0.8

*BMI* body mass index, *M–L* medial–lateral distance, *A–P* the maximum anterior–posterior distance at the medial compartment, and *JS* joint space measured in the medial compartment

#### Finite Element Models

We made assumptions and simplifications aiming for the rapid simulation of subject-specific knee models under a gait loading condition. Regarding the geometries, FE models comprised the cartilages of the medial compartment of the tibiofemoral joint [26] since knee OA is more frequent in the medial than in the lateral compartment [3, 19]. We did not include ligaments, tendons, and muscles to avoid increasing computational costs [7]. However, their constraining effects were considered through appropriate but simplified boundary conditions, by fixing anterior–posterior and medial–lateral translations, and internal–external rotations. The meniscus was excluded from the simulations, but its contribution was accounted for indirectly by subtracting the meniscus support force from the force acting through the tibiofemoral joint [26]. This assumption conveys similar stress and fluid pressure distributions in cartilage–cartilage contact regions during walking as if we would physically consider the meniscus in the model [26]. Regarding loading, we prescribed generic effective joint axial force and knee flexion over an analytical point located at the mid-distance between the epicondyles of the distal femur. We assumed the medial compartment of the knee bears half of the joint reaction force during gait. This is because we did not account for the individual motion data of the subjects, and this assumption fits into the experimental observations for the medial/lateral load sharing [22, 43].

#### Template Model Scaling

We followed the method proposed by Mononen et al. (2019). We scaled the FE mesh of one existing template based on the fractional differences in M–L, A–P, and JS parameters between the subject of interest and the template (Fig. 1). A cartesian scaling was used for anatomical dimensions, except for femoral cartilage thickness, where a cylindrical scaling was utilized. Regarding loading, we scaled the axial joint contact force [23] based on the body weight of each subject and kept the knee flexion angle trajectory [6] the same for all models.

#### Framework Comparisons (ABAQUS vs FEBio)

Finite element software helps model complex problems involving multiphasic physics in biomechanics. ABAQUS (Dassault Systèmes Simulia Corp., Providence, RI) is a well-known commercial software in computational biomechanics with useful characteristics for researchers, like user-defined functions for material formulations and boundary conditions. On the other hand, FEBio is a freely available option for solving these non-linear biphasic problems [25]. In this regard, we aimed at exploring the transferability of the template-based approach using both of these highly known programs.

In a previous study, we showed how to impose equivalent boundary conditions and similar constitutive equations in a knee joint model using both ABAQUS and FEBio softwares [33]. Regarding cartilage material, in ABAQUS standard v.2018, we used an experimentally validated fibril-reinforced poroviscoelastic formulation [15, 41, 42], while in FEBio 3.3.2 we used a fibril-reinforced biphasic description [5, 33], with material properties obtained to reproduce the instantaneous response of the material used in ABAQUS. In both frameworks, we implemented the same depth-dependent orientation for collagen fibrils. Table 2 summarizes the cartilage model parameters.

**Table 2:** Cartilage material properties implemented in FEBio and ABAQUS

Cartilage model	Property	Tibia	Femur	Model representation
Fibril-reinforced poroviscoelastic (ABAQUS)	${E}_{e}$ (MPa)^a^	23.63	150	
	${E}_{0}$(MPa)^a^	0.18	0.92	
	*C*(−)^a^	12.16	12.16	
	$\eta$(MPa s)^a^	1062	1062	
Biphasic fibril-reinforced (FEBio)	${\xi }_{fp}$(MPa)^b^	32	215	
	${\beta }_{fp}$(−)^b^	2.6	2.6	
	${\xi }_{fs}$(MPa)^b^	1.0	3.0	
	${\beta }_{\mathrm{fs}}$(−)^b^	2.6	2.6	
Properties in common	${E}_{nf}$(MPa)^a^	0.106	0.215	
	${\nu }_{nf}$(−)^a^	0.15	0.15	
	${\kappa }_{0}$(10^–15^ m^4^ N^−1^ s^−1^)^a^	18	6	
	*M*(−)^a^	15.24	5.06	
	${\varphi }_{0}$(−)^c^	0.2	0.2	

${E}_{e}$ is the strain-dependent elastic modulus of fibrils, ${E}_{0}$, linear elastic stiffness of fibrils, *C* ratio of primary to secondary fibrils, $\eta$ damping coefficient of fibrils, ${\xi }_{fp}$, stiffness parameter of primary fibrils, ${\xi }_{fs}$ stiffness parameter of secondary fibrils, ${\beta }_{fp}$ is the power of primary fibrils, ${\beta }_{fs}$ is the power of secondary fibrils, ${E}_{nf}$ is Young’s modulus of the isotropic ground matrix and ${v}_{nf}$ its Poisson’s ratio, ${\kappa }_{0}$ initial hydraulic permeability, *M* the material parameter for strain-dependent models of permeability, and ${\varphi }_{0}$ initial solid fraction

^a^Julkunen et al. (2007)

^b^Paz et al. (2022)

^c^Holmes and Mow (1990)

We compared the results of both finite element softwares in terms of time and space. These comparisons include average and peak values over the contact area, stress maps at different fractions of the stance, and volumes of tissue overstressed for different KL grade groups. The “Statistical Analysis” section describes these comparisons.

#### Sensitivity Analysis

We performed a factorial design to assess the sensitivity of the peak tensile stress occurring at the first load peak of the stance phase of gait with respect to the parameters used to generate the compartment models. This is because our degeneration model relies on age-dependent thresholds of cartilage tensile stress to define the volume of tissue at risk (see “Damage Model” section). The four factors were the weight of the subject and measurements of knee M–L distance, medial knee A–P distance, and medial JS width. The low and high levels of these factors were the minimum and maximum values observed in the sample, respectively. Table 3 summarizes the 16 combinations of the four factors and the two levels, e.g., model 1 was constructed using the high level for all parameters.

**Table 3:** Level distributions across the 16 models of the factorial design

Model	1	2	3	4	5	6	7	8	9	10	11	12	13	14	15	16
Weight	+	+	+	+	+	+	+	+	−	−	−	−	−	−	−	−
M-L	+	+	+	+	−	−	−	−	+	+	+	+	−	−	−	−
A-P	+	+	−	−	+	+	−	−	+	+	−	−	+	+	−	−
JS	+	−	+	−	+	−	+	−	+	−	+	−	+	−	+	−

(+) high level, (−) low level

*M–L* medial–lateral measurement, *A–P* anterior–posterior measurement, and *JS* medial joint space measurement

To assess the sensitivity of the peak stress with respect to each factor, we first calculated the overall mean peak stress from the 16 models ($\overline{Peak}$). Then, we calculated the mean peak stresses from the eight models sharing either the highest ($+$) or the lowest (−) level $i$ of each factor $j$, e.g., $Pea{k}_{+,j}$ or $Pea{k}_{-,j}$ for the high and low levels, respectively. Finally, we computed the relative difference of each of these peaks with respect to the overall mean peak stress,

1 $${\text{Relative}}\,{\text{difference}}_{i,j} = \frac{{\left( {Peak_{i,j} - \overline{Peak} } \right)}}{{\overline{Peak} }},$$

$$ \begin{aligned} & i = \left\{ { - , + } \right\}, j = \left\{ {{\text{Weight}}, M - L,A - P,JS} \right\}, \\ & Peak_{i,j} = {\text{mean}}\left( {{\text{Stress }} {\text{peaks }} {\text{from }} {\text{the }} 8 {\text{ models }} {\text{sharing }} {\text{the }} {\text{level }} i {\text{ of }} {\text{the }} {\text{factor }} j} \right), \\ & \overline{Peak} = {\text{mean}}\left( {{\text{Stress }} {\text{peaks }} {\text{from }} {\text{the }} 16 {\text{ models}}}\right).\end{aligned}$$

### Damage Model

We used tensile stress thresholds to define tissue degeneration. In this way, we assume the damage is initiated in the fibril collagen network since this constituent is mainly in charge of the tensile stresses [14, 21]. We considered the threshold for damage (${T}_{{\sigma }_{f}})$ according to experimental observations of tensile tests of human articular cartilage over a wide range of ages [17, 26]. Then, ${T}_{{\sigma }_{f}}$ was defined as,

2a $$T_{{\sigma_{f} }} = 30 {\text{ MPa}},\quad {\text{Age}} \le 30$$

2b $$T_{{\sigma_{f} }} = \left( {30 - \left( {{\text{Age}} - 30} \right)\left( {\frac{20}{{15}}} \right) } \right) {\text{MPa}},\quad 30 < {\text{Age}} \le 45$$

2c $$T_{{\sigma_{f} }} = \left( {10 - \left( {{\text{Age}} - 45} \right)\left( \frac{3}{20} \right) } \right) {\text{MPa}},\quad 45 < {\text{Age}} \le 65$$

2d $$T_{{\sigma_{f} }} = \left( {7 - \left( {{\text{Age}} - 65} \right)\left( \frac{2}{100} \right) } \right) {\text{MPa}},\quad 65 < {\text{Age}} \le 75$$

2e $$T_{{\sigma_{f} }} = 6.8\,{\text{MPa}},\quad {\text{Age}} > 75.$$

Subsequently, we hypothesized that larger volumes of overstressed tissue positively correlate with a higher risk of developing knee OA. Thus, in our analyses, the volume of tissue at risk was equal to the volume occupied by the elements that exceeded the thresholds during the simulated loading cycle.

### Classification Capabilities

We tested our method's ability to distinguish future osteoarthritic knees from healthy knees using simulated degenerated tissue volumes. To this end, we modeled the knees at the healthy baseline (KL0,1) and computed the volumes at risk. We then grouped the volumes using the KL grades determined in the 8th year of follow-up and reported the area under the curve (AUC) of receiver operating characteristic (ROC) curves between pairs of KL grades.

### Statistical Analysis

We compared the results of two finite element softwares as well as the volumes of overstressed cartilage from the different KL groups of simulated knees.

To compare the stress results of both FE softwares, we used one-dimensional (1D) and two-dimensional (2D) statistical parametric mapping (SPM{t}). [31] In 1D statistical analyses, we compared the average and peak values of the maximum principal stress over the tibial contact area during the load cycle. We used the two-tailed 1D nonparametric SPM{t} (*α* = 0.05) since these variables did not follow normal distributions. In 2D statistical analyses, we compared the spatial stress distributions in the superficial cartilage layer for different time points of the stance fraction. For that, we staked the histories of stress maps from FEBio and ABAQUS models and used the two-tailed 2D nonparametric SPM{t} (*α* = 0.05). This was possible because we used the same meshes and matched the time points between the softwares.

Regarding the comparison of the volumes of overstressed tissue from each software for the same KL groups, we used the Wilcoxon signed-rank test. To compare the volumes of overstressed tissue between KL groups, we used Kruskal–Wallis’s test followed by Dunn’s test. All the analyses were implemented in MATLAB R2019b (The MathWorks, Natick, MA, USA).

## Results

**Fig. 3:**
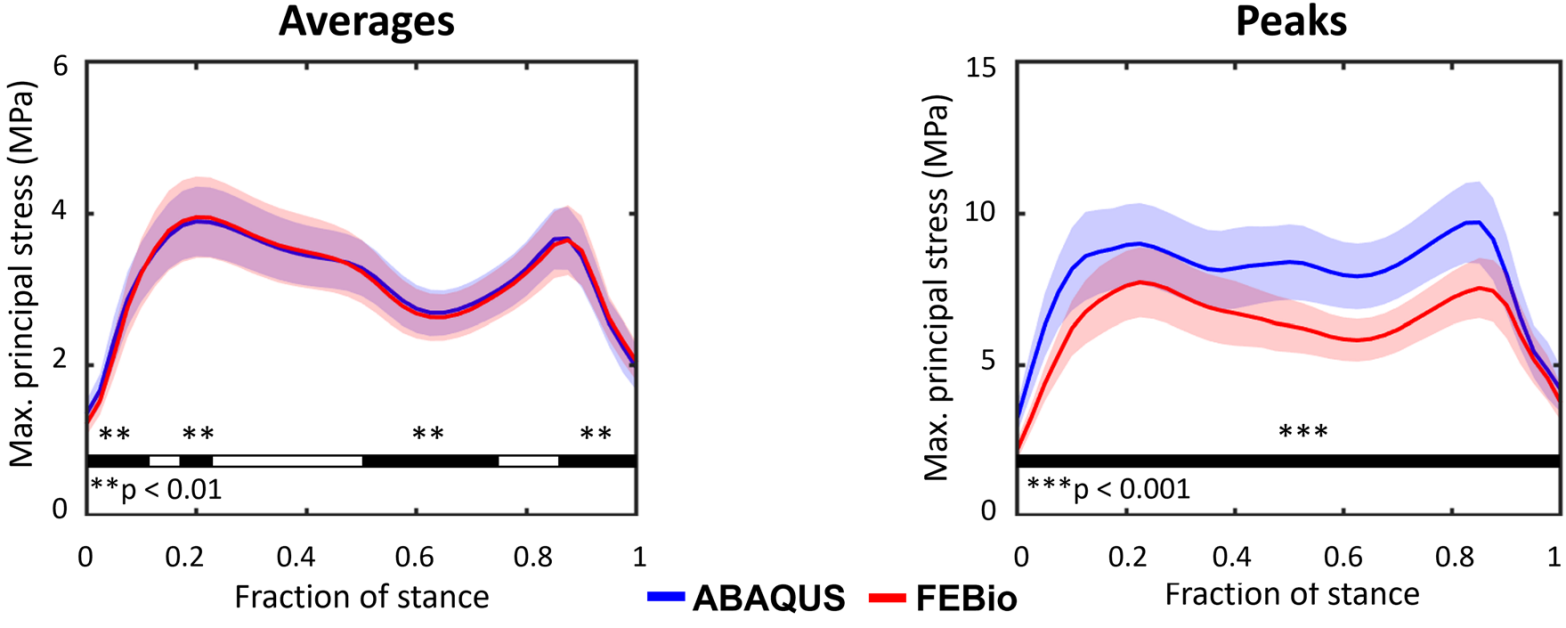
Average (left) and peak (right) time distributions of the maximum principal stress over the tibial contact area. Solid lines represent the mean value and shaded regions represent one standard deviation. The bottom thick lines indicate statistically significant differences in distributions obtained by 1D nonparametric SPM

**Fig. 4:**
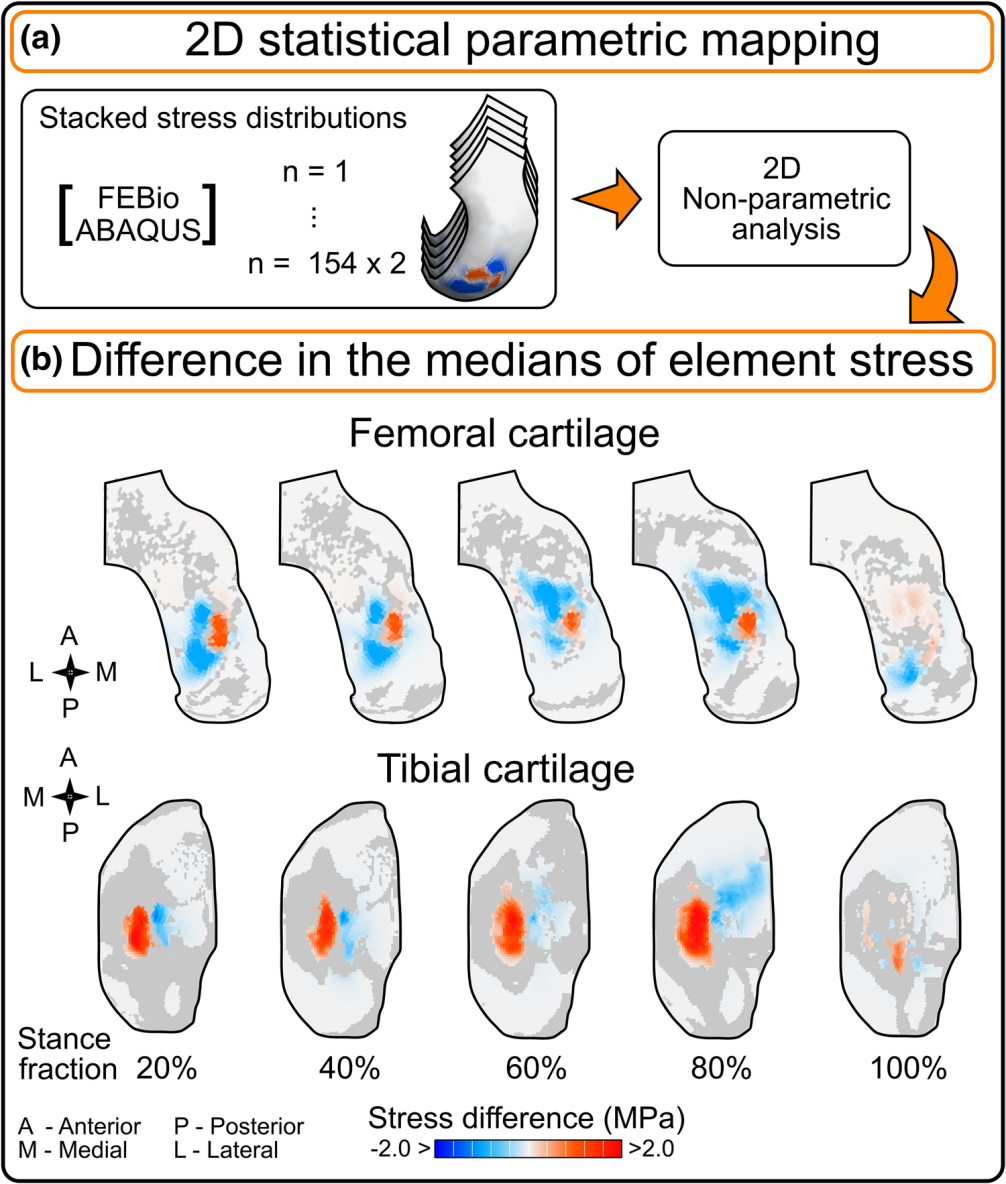
Contours of difference in the medians of the maximum principal stress maps between ABAQUS and FEBio models. The contours depicted correspond to the superficial cartilage layer. ${\text{Stress}}\,{\text{difference}}=median\,\left(Stres{s}_{ABAQUS}-Stres{s}_{FEBio}\right).$. Colored regions show the elements where 2D SPM{t} suggested statistically significant differences (*α* = 0.05). Gray zones indicate regions where the stress distributions did not differ

The temporal and spatial distributions of the maximum principal stress showed some variation that was dependent on the software that was utilized. While the principal stress averaged over the contact area was essentially the same in both programs (Fig. 3), the stress peaks during the load cycle using ABAQUS were on average 27% higher than those from FEBio (Fig. 3). Comparisons for maximum principal strain and fluid pressure can be found in Section 2a of the **Supplementary Material**. Regarding the spatial distribution, Fig. 4 highlights the regions where the maximum principal stress statistically differed between softwares at different stance fractions. The red zones indicate that ABAQUS offered higher values compared to FEBio, and the blue zones indicate the opposite trend. The gray color depicts regions with no differences. In Section 2b of the Supplementary Material, the reader can appreciate the influence of the scaling geometry parameters (M–L, A–P, JS) on the differences between programs.

**Fig. 5:**
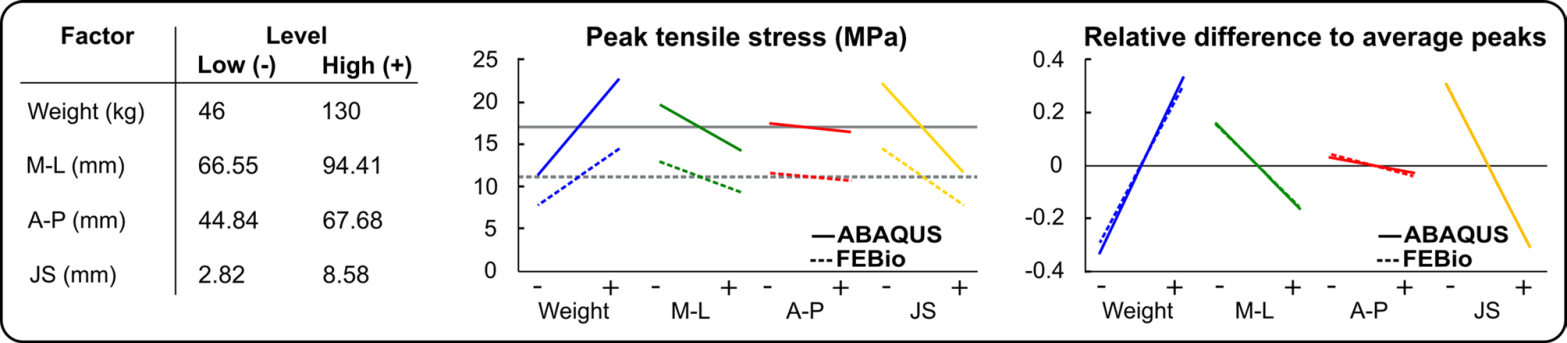
Sensitivity of peak maximum principal stress at 20% of the stance fraction of gait to low and high levels of the four factors (left) used to generate the compartment models. Plots show absolute stress values (center) and relative differences from using Eq. 1 (right), grouped by each factor, for FEBio and ABAQUS implementations. Medial–lateral (M–L) distance, maximum anterior–posterior (A–P) distance, and medial joint space (JS) width

Regarding the sensitivity analysis, Fig. 5 shows higher peak tensile stresses in ABAQUS models compared to FEBio models. Both FE software implementations of the template-based approach yielded similar relative sensitivity of stresses to the parameters used to generate the models. The weight and joint space (JS) were the factors that had the greatest influence on the peak stress, with the steepest slopes, as shown in Fig. 5. In contrast, the anterior–posterior distance (A–P) caused a minimal variation in the peak stress between the low and high levels.

**Fig. 6:**
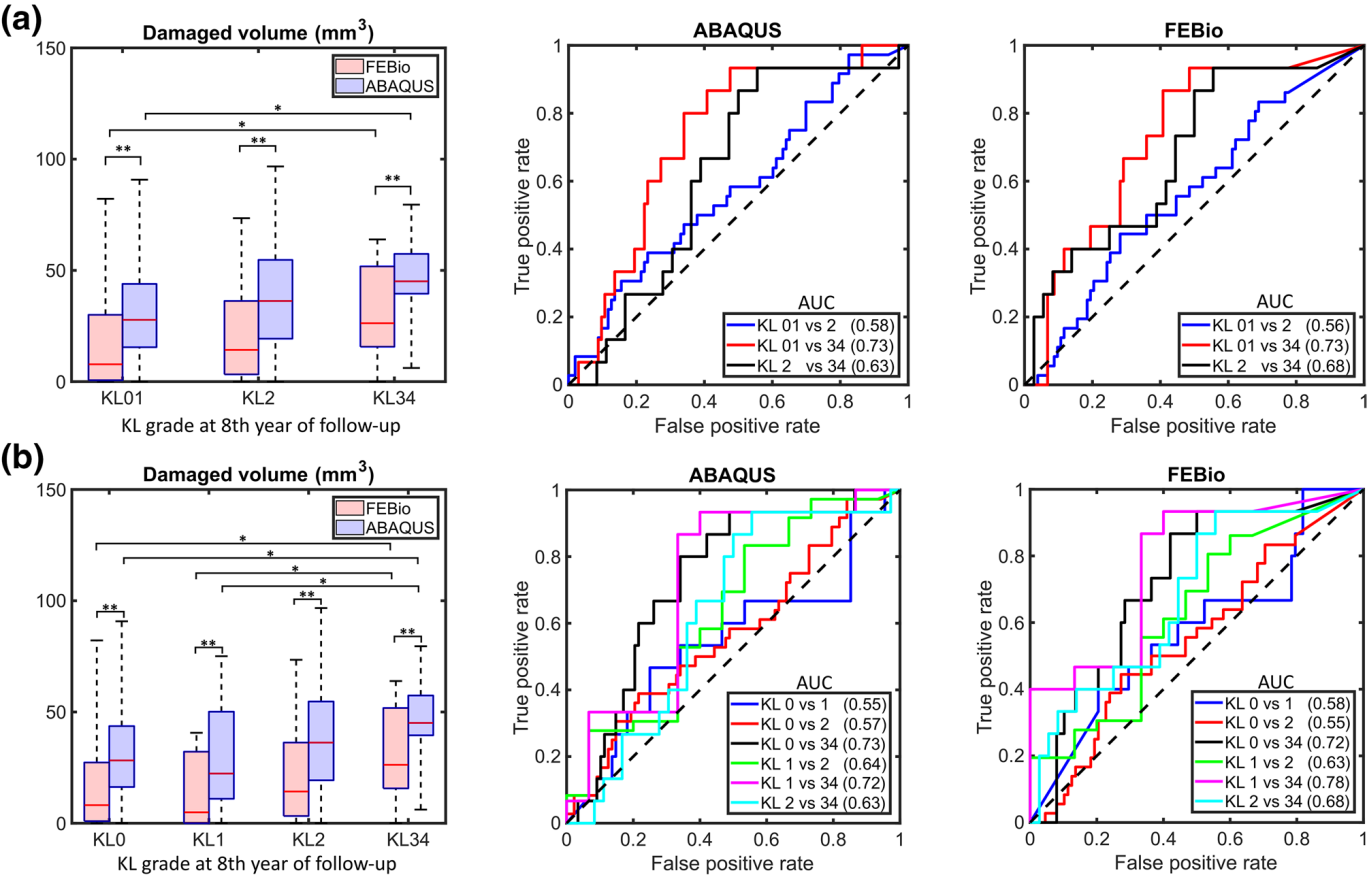
Volume comparisons and ROC curves between KL grades using ABAQUS and FEBio frameworks. **a** Classification using 3 KL grades and **b** using 4 KL grades. Statistical significance was evaluated using Kruskal–Wallis and Dunn’s tests between KL grades, and the Wilcoxon rank test for the same KL grades. *p < 0.05, **p < 0.01

Regarding the simulated degenerated volumes, for the corresponding KL groups, the ABAQUS volumes were about 43% greater than those from FEBio (Fig. 6). Nevertheless, both softwares yielded significant differences between healthy (KL0,1) and severely affected (KL3,4) knees, when the knees were separated into three or four groups (Fig. 6).

**Fig. 7:**
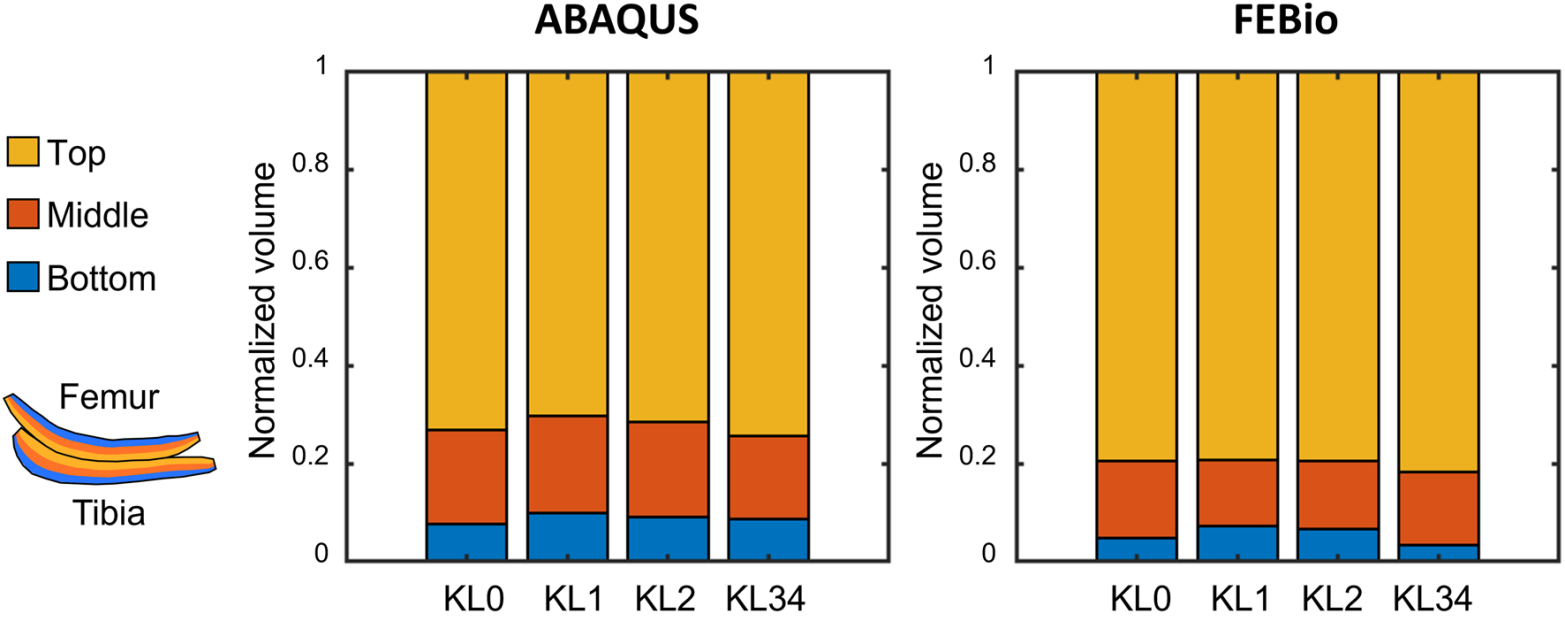
Normalized volumes of overstressed cartilage for the different KL grades indicate how each tissue layer contributes to the total volumes

Finally, looking at the layers of the overstressed tissue (Fig. 7), the superficial zone is responsible for ~ 80% of these volumes, while the middle zone contributes ~ 15% and the bottom zone contributes ~ 5%. These proportions remained constant between KL groups, independently of the software utilized.

## Discussion

A template-based finite element approach was implemented in commercial (ABAQUS) and freely available (FEBio) FE software to generate knee joint models of 154 healthy knees. We used the simulated levels of maximum principal stresses in the knees to predict personalized cartilage degeneration. The predicted volumes of degenerated tissue were compared against KL grade changes after an 8-year follow-up. Though there were notable differences in the simulated absolute values for the maximum principal stresses, similar OA classification outcomes were predicted for the healthy and osteoarthritic knee joints with both softwares based on the ROC analysis. This suggests that simpler constitutive models for cartilage may be used to simulate OA degeneration. In addition, we investigated the contributions of subject-specific factors (joint shape and weight) on the simulated peak stresses in cartilage, as this is utilized to generate a prediction for cartilage degeneration as a function of age. From this analysis, we found that peak stresses in both software implementations were similarly sensitive to the parameters used to generate the models.

Results showed notable differences in the tensile stress over the tibiofemoral contact area during the gait loading between softwares. The stress distributions showed regions where either ABAQUS or FEBio simulated higher values. These differences may arise from the different material formulations between ABAQUS and FEBio. In ABAQUS, we implemented an FRPVE cartilage model that we mimicked with a fibril-reinforced poroelastic cartilage model in FEBio [33]. Therefore, the variability in loading conditions with respect to those used to calibrate the FEBio material properties exposes this difference in formulations.

The sensitivity analysis indicates that the peak of maximum principal stress was similarly sensitive to the anatomical and loading factors used in the template-based FE modeling in both softwares. This finding supports the idea that the method could work as a means for studying these biomechanical risk factors and translating them into quantifiable modeled variables such as stress or volumes of tissue at risk for the onset of OA development. Weight positively influenced peak stress, indicating that being overweight increases the chances of the tissue failing, which agrees with known biomechanical knee OA risks [3]. On the other hand, the JS negatively influences the peak stress, suggesting that thinning of tissue also increases the stress when it bears the same weight compared to a control sample, which is in agreement with previous studies correlating biomechanics and tissue adaptation or damage [4, 29]. The medial–lateral (M–L) and anterior–posterior (A–P) distances showed a lower influence compared to weight and joint space. However, we considered that M–L and A–P dimensions may affect the congruency of the joint and may have a greater influence if the loading involves larger relative rotations of bone ends, like in deep squatting [22].

Regarding the predictions of cartilage degeneration, we found that the simulated volumes of overstressed tissue differed between software implementations. Although, both cases acceptably classified healthy (KL01) from severely affected joints (KL34), suggested by an AUC ~ 0.73. In addition, despite the promising difference observed between KL1 and KL3,4, using 4 KL groups, the proposed approach did not show differences between KL0 and KL1 groups. It should be noted that KL1 does not indicate degenerative changes in cartilage, but rather doubtful joint space narrowing (and possible osteophytic lipping) [20], which can be interpreted as normal joint space in many cases. Therefore, it might be an unrealistic goal to develop a method that could predict future joint conditions between KL0 and KL1.

We based our predictions on the short-term biomechanical response of the collagen network, simulated by the maximum principal stresses (tensile stresses). However, other failure criteria and loading conditions should be explored for predicting the onset and progression of cartilage damage with cohort data. For instance, the fatigue behavior of cartilage under tension [39, 40], shear, compression [16], or sliding [9], as well as tissue remodeling induced by the biochemical signaling of cells [28, 36]. Combining these damage mechanisms may yield more accurate physics-based computational approaches to determine not only the severity but the location and pathway of tissue damage. Although, validating such models is challenging since it is hard to obtain suitable experimental measurement data to compare with, especially when using knee-level models.

Figure 7 revealed that most of the volume of overstressed tissue is located in the superficial layer of cartilage. This distribution is explained by the fibril-reinforced formulation and the depth-dependent orientation of the collagen network. In addition, this finding suggests that, for predictive purposes, focusing our attention only on the cartilage surface may represent a saving in computational time. In this regard, using contact stress from discrete element analysis (DEA) [1, 38] seems to be a possibility. DEA approaches have been evaluated in predicting the future stages of knee OA [35] and ankle post-traumatic OA [18] with promising results. Although we acknowledge that this method is not appropriate to study structure–function alterations in the tissue.

The time required for simulating the 154 knee models was 34 h in FEBio and 123 h in ABAQUS. This difference in time is explained by the differences in material model formulations between ABAQUS and FEBio, and the limited parallelization allowed by the licensing of ABAQUS. Although the original cartilage degeneration algorithm was developed with ABAQUS using the FRPVE material formulation for cartilage [26], the use of FEBio is also recommended based on the similar classification accuracy (KL01 vs KL34 knees) we observed. In FEBio, users can run as many models as computing power allows, while in ABAQUS, users are limited by the number of licenses (tokens) purchased. The benefit of using FEBio also offers a more cost-effective route to prediction results with large datasets.

The limitations of the present work include the absence of the lateral tibiofemoral compartment, restricting the identification of regions at risk to the medial compartment. In addition, we assumed a fixed load distribution between medial and lateral compartments, since the Osteoarthritis Initiative database does not have motion data. We assumed a scaled generic knee loading and a fixed flexion trajectory, limiting the study of the effects of subject-specific gait patterns that may overload or protect different contact regions in the knee. We did not use subject-specific material properties in the joint, which may show the site-specific onset of OA [24]. Another characteristic that we consider a limitation was not including local morphologies in the cartilage or subchondral bone that can induce the onset and progression of damage [37], making the scaling of the template geometry sound simplistic. However, in the original paper by Mononen et at. 2019, they compared 21 knee models using the actual subject-specific geometries, obtained from manual segmentations, and scaling templates. They found that the model with the scaled geometries produced better results. Therefore, we did not consider other solutions to implement template scaling. We additionally consider that the low number of OA knees can be a limitation if we aim to calibrate predictive models using, for instance, machine learning tools. Furthermore, we compared only two softwares to give insights into the transferability of the method. In this case, it would be worth exploring the reproducibility not only between softwares but also among researchers. [10, 34]

In the future, we will implement our template-based approach to consider the lateral compartment of the tibiofemoral joint. This improvement should include the geometry of the lateral compartment and the differentiated medial/lateral loading during the stance phase of gait. Moreover, we will explore the capability of other biomechanical responses, such as the maximum shear strain, to identify the future knee OA stage, as they have been associated with damage in other tissue constituents than the collagen network [14]. Finally, considering local defects in the models can be a valuable feature to expand the use of our approach in cohort studies of post-traumatic knee OA. This could be done by rapidly defining affected zones by imposing adequate boundary conditions and depleting the mechanical properties of the tissue (e.g., low elastic properties, high permeability), as well as by performing proper local mesh refinement.

In conclusion, the template-based method to simulate the biomechanical behavior of the medial compartment of the knee was extrapolated to two widely used FE softwares obtaining similar acceptable classifications of the future knee KL grades. Through this work, we encourage the development of reproducible methods to study and predict the degeneration of human body structures, aiming for tools to help clinicians elucidate conservative treatments for multifactorial musculoskeletal disorders.

### Supplementary Information

Below is the link to the electronic supplementary material.

Supplementary file1 (PDF 562 KB)

## References

[1] Aitken HD, Westermann RW, Bartschat NI, Meyer AM, Brouillette MJ, Glass NA, Clohisy JC, Willey MC, Goetz JE (2022). Chronically elevated contact stress exposure correlates with intra-articular cartilage degeneration in patients with concurrent acetabular dysplasia and femoroacetabular impingement. J. Orthop. Res..

[2] Ali AA, Shalhoub SS, Cyr AJ, Fitzpatrick CK, Maletsky LP, Rullkoetter PJ, Shelburne KB (2016). Validation of predicted patellofemoral mechanics in a finite element model of the healthy and cruciate-deficient knee. J. Biomech..

[3] Allen KD, Golightly YM (2015). Epidemiology of osteoarthritis: state of the evidence. Curr. Opin. Rheumatol..

[4] Andriacchi TP, Briant PL, Bevill SL, Koo S (2006). Rotational changes at the knee after ACL injury cause cartilage thinning. Clin. Orthop. Related Res..

[5] Ateshian GA, Rajan V, Chahine NO, Canal CE, Hung CT (2009). Modeling the matrix of articular cartilage using a continuous fiber angular distribution predicts many observed phenomena. J. Biomech. Eng..

[6] Bergmann G, Bender A, Graichen F, Rohlmann A, Trepczynski A, Heller MO, Kutzner I (2014). Standardized loads acting in knee implants. PLoS ONE.

[7] Bolcos PO, Mononen ME, Mohammadi A, Ebrahimi M, Tanaka MS, Samaan MA, Souza RB, Li X, Suomalainen JS, Jurvelin JS, Töyräs J, Korhonen RK (2018). Comparison between kinetic and kinetic-kinematic driven knee joint finite element models. Sci. Rep..

[8] Bolcos PO, Mononen ME, Tanaka MS, Yang M, Suomalainen J-S, Nissi MJ, Töyräs J, Ma B, Li X, Korhonen RK (2020). Identification of locations susceptible to osteoarthritis in patients with anterior cruciate ligament reconstruction: Combining knee joint computational modelling with follow-up T1ρ and T2 imaging. Clin. Biomech..

[9] Durney KM, Shaeffer CA, Zimmerman BK, Nims RJ, Oungoulian S, Jones BK, Boorman-Padgett JF, Suh JT, Shah RP, Hung CT, Ateshian GA (2020). Immature bovine cartilage wear by fatigue failure and delamination. J. Biomech..

[10] Erdemir A, Besier TF, Halloran JP, Imhauser CW, Laz PJ, Morrison TM, Shelburne KB (2019). Deciphering the “art” in modeling and simulation of the knee joint: Overall strategy. J. Biomech. Eng..

[11] Gardiner BS, Woodhouse FG, Besier TF, Grodzinsky AJ, Lloyd DG, Zhang L, Smith DW (2016). Predicting knee osteoarthritis. Ann. Biomed. Eng..

[12] Henak CR, Anderson AE, Weiss JA (2013). Subject-specific analysis of joint contact mechanics: Application to the study of osteoarthritis and surgical planning. J. Biomech. Eng..

[13] Henak CR, Carruth ED, Anderson AE, Harris MD, Ellis BJ, Peters CL, Weiss JA (2013). Finite element predictions of cartilage contact mechanics in hips with retroverted acetabula. Osteoarthr. Cartil..

[14] Hosseini SM, Wilson W, Ito K, Van Donkelaar CC (2014). A numerical model to study mechanically induced initiation and progression of damage in articular cartilage. Osteoarthr. Cartil..

[15] Julkunen P, Kiviranta P, Wilson W, Jurvelin JS, Korhonen RK (2007). Characterization of articular cartilage by combining microscopic analysis with a fibril-reinforced finite-element model. J. Biomech..

[16] Kaplan JT, Neu CP, Drissi H, Emery NC, Pierce DM (2017). Cyclic loading of human articular cartilage: The transition from compaction to fatigue. J. Mech. Behav. Biomed. Mater..

[17] Kempson GE (1982). Relationship between the tensile properties of articular cartilage from the human knee and age. Ann. Rheum. Dis..

[18] Kern AM, Anderson DD (2015). Expedited patient-specific assessment of contact stress exposure in the ankle joint following definitive articular fracture reduction. J. Biomech..

[19] Khan HI, Aitken D, Chou L, McBride A, Ding C, Blizzard L, Pelletier JP, Pelletier JM, Cicuttini F, Jones G (2015). A family history of knee joint replacement increases the progression of knee radiographic osteoarthritis and medial tibial cartilage volume loss over 10 years. Osteoarthr. Cartil..

[20] Kohn MD, Sassoon AA, Fernando ND (2016). Classifications in Brief: Kellgren–Lawrence classification of osteoarthritis. Clin. Orthop. Relat. Res..

[21] Korhonen RK, Laasanen MS, Töyräs J, Lappalainen R, Helminen HJ, Jurvelin JS (2003). Fibril reinforced poroelastic model predicts specifically mechanical behavior of normal, proteoglycan depleted and collagen degraded articular cartilage. J. Biomech..

[22] Kutzner I, Bender A, Dymke J, Duda G, Von Roth P, Bergmann G (2017). Mediolateral force distribution at the knee joint shifts across activities and is driven by tibiofemoral alignment. Bone Jt. J..

[23] Kutzner I, Heinlein B, Graichen F, Bender A, Rohlmann A, Halder A, Beier A, Bergmann G (2010). Loading of the knee joint during activities of daily living measured in vivo in five subjects. J. Biomech..

[24] Lampen N, Chan DD (2022). Finite element modeling with subject-specific mechanical properties to assess knee osteoarthritis initiation and progression. J. Orthop. Res..

[25] Maas SA, Ellis BJ, Ateshian GA, Weiss JA (2012). FEBio: Finite elements for biomechanics. J. Biomech. Eng..

[26] Mononen ME, Liukkonen MK, Korhonen RK (2019). Utilizing atlas-based modeling to predict knee joint cartilage degeneration: Data from the osteoarthritis initiative. Ann. Biomed. Eng..

[27] Mukherjee S, Nazemi M, Jonkers I, Geris L (2020). Use of computational modeling to study joint degeneration: A review. Front. Bioeng. Biotechnol..

[28] Neidlin M, Chantzi E, Macheras G, Gustafsson MG, Alexopoulos LG (2019). An ex vivo tissue model of cartilage degradation suggests that cartilage state can be determined from secreted key protein patterns. PLoS ONE.

[29] Das Neves Borges P, Forte AE, Vincent TL, Dini D, Marenzana M, Das Neves P, Forte AE, Vincent TL, Dini D, Marenzana M (2014). Rapid, automated imaging of mouse articular cartilage by microCT for early detection of osteoarthritis and finite element modelling of joint mechanics. Osteoarthr. Cartil..

[30] Orozco GA, Bolcos P, Mohammadi A, Tanaka MS, Yang M, Link TM, Ma B, Li X, Tanska P, Korhonen RK (2021). Prediction of local fixed charge density loss in cartilage following ACL injury and reconstruction: A computational proof-of-concept study with MRI follow-up. J. Orthop. Res..

[31] Pataky TC (2010). Generalized n-dimensional biomechanical field analysis using statistical parametric mapping. J. Biomech..

[32] Paz A, Orozco GA, Korhonen RK, García JJ, Mononen ME (2021). Expediting finite element analyses for subject-specific studies of knee osteoarthritis: A literature review. Appl. Sci..

[33] Paz A, Orozco GA, Tanska P, García JJ, Korhonen RK, Mononen ME (2022). A novel knee joint model in FEBio with inhomogeneous fibril-reinforced biphasic cartilage simulating tissue mechanical responses during gait: data from the osteoarthritis initiative. Comput. Methods Biomech. Biomed. Engin..

[34] Rooks NB, Schneider MTY, Erdemir A, Halloran JP, Laz PJ, Shelburne KB, Hume DR, Imhauser CW, Zaylor W, Elmasry S, Schwartz A, Chokhandre SK, Abdollahi Nohouji N, Besier TF (2021). Deciphering the “art” in modeling and simulation of the knee joint: Variations in model development. J. Biomech. Eng..

[35] Segal NA, Anderson DD, Iyer KS, Baker J, Torner JC, Lynch JA, Felson DT, Lewis CE, Brown TD (2009). Baseline articular contact stress levels predict incident symptomatic knee osteoarthritis development in the MOST cohort. J. Orthop. Res..

[36] Shim VB, Hunter PJ, Pivonka P, Fernandez JW (2011). A multiscale framework based on the Physiome markup languages for exploring the initiation of osteoarthritis at the bone-cartilage interface. IEEE Trans. Biomed. Eng..

[37] Teichtahl AJ, Wluka AE, Wang Y, Forbes A, Davies-Tuck ML, English DR, Giles GG, Cicuttini FM (2012). Effect of long-term vigorous physical activity on healthy adult knee cartilage. Med. Sci. Sports Exerc..

[38] Volokh KY, Chao EYS, Armand M (2007). On foundations of discrete element analysis of contact in diarthrodial joints. MCB Mol. Cell. Biomech..

[39] Weightman B (1976). Tensile fatigue of human articular cartilage. J. Biomech..

[40] Weightman B, Chappell DJ, Jenkins EA (1978). A second study of tensile fatigue properties of human articular cartilage. Ann. Rheum. Dis..

[41] Wilson W, Van Donkelaar CC, Van Rietbergen B, Ito K, Huiskes R (2004). Stresses in the local collagen network of articular cartilage: A poroviscoelastic fibril-reinforced finite element study. J. Biomech..

[42] Wilson W, Van Donkelaar CC, Van Rietbergen B, Ito K, Huiskes R (2005). Erratum: A fibril-reinforced poroviscoelastic swelling model for articular cartilage (Journal of Biomechanics (2005) 38 (1195–1204) PII: S0021929004003367 and DOI: 10.1016/S0021-9290(03)00267-7). J. Biomech..

[43] Zhao D, Banks SA, Lima DDD, Colwell CW, Fregly BJ (2007). In vivo medial and lateral tibial loads during dynamic and high flexion activities. J. Orthop. Res..

