# Supplementary material for: Towards a Transferable Modeling Method of the Knee to Distinguish Between Future Healthy Joints from Osteoarthritic Joints: Data from the Osteoarthritis Initiative
Source: Ann Biomed Eng. 2023 Jun 7;51(10):2192–203. doi: 10.1007/s10439-023-03252-8 (PMC10518288; doi:10.1007/s10439-023-03252-8)
Supplement: Supplementary file 1 — Supplementary file1 (PDF 562 KB) [file 10439_2023_3252_MOESM1_ESM.pdf]

Supplementary material for:

Towards a transferable modeling method for the knee to distinguish between future healthy joints from osteoarthritic joints: data from the Osteoarthritis Initiative

Alexander Paz[1,2], José Jaime García[2], Rami Korhonen[1], Mika Mononen[1]

1 Department of Technical Physics, University of Eastern Finland, Kuopio, Finland

2 Escuela de Ingeniería Civil y Geomática, Universidad del Valle, Cali, Colombia

alexander.paz@uef.fi

Yliopistonranta 1, 70211, Kuopio, Finland

1. Methods

a. Subject characteristics

Fig. S1 shows box plots of the characteristics of the subjects. This figure indicates there is only a significant difference in the distribution of the age of the KL1 group compared to the others. In addition, there are no differences in the other parameters between any pair of groups.

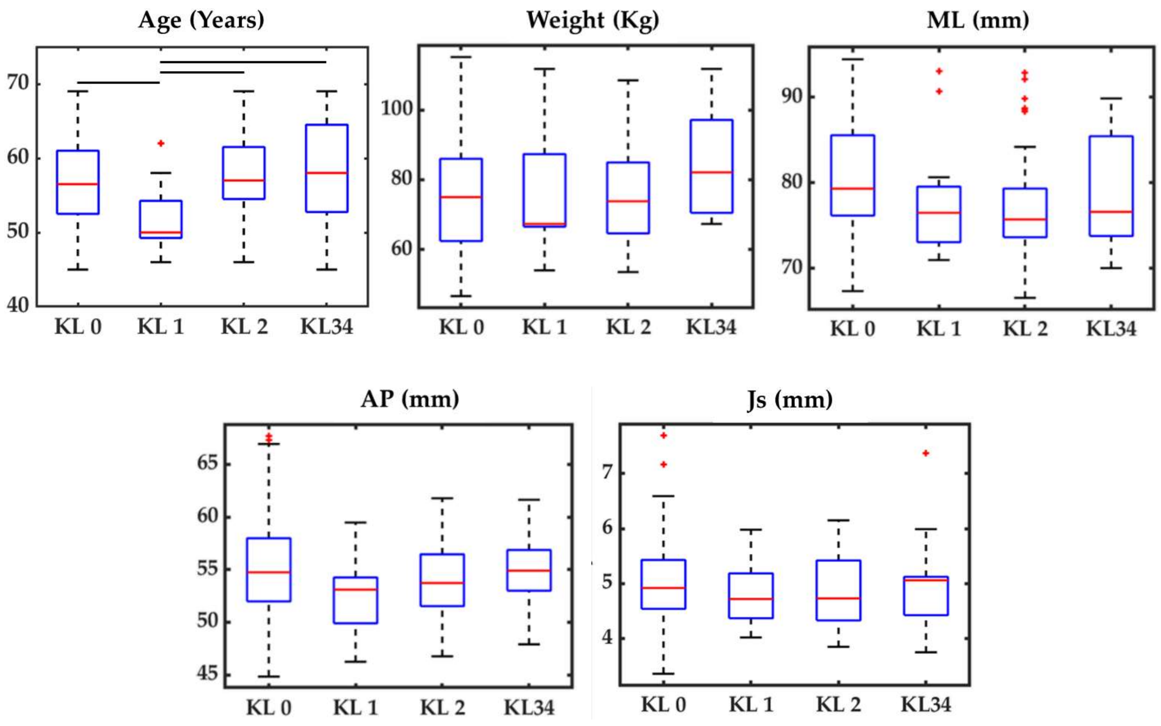

Figure S1. Summary of subject characteristics at the baseline: Age, weight, medial-lateral distance (ML), anterior-posterior distance (AP), and joint space distance (JS).

2. Results

a. Differences 1D SPM

Fig. S2 shows the differences observed in fluid pressure and maximum principal strain. This figure indicates that the averages and peak values obtained from the different approaches differ.

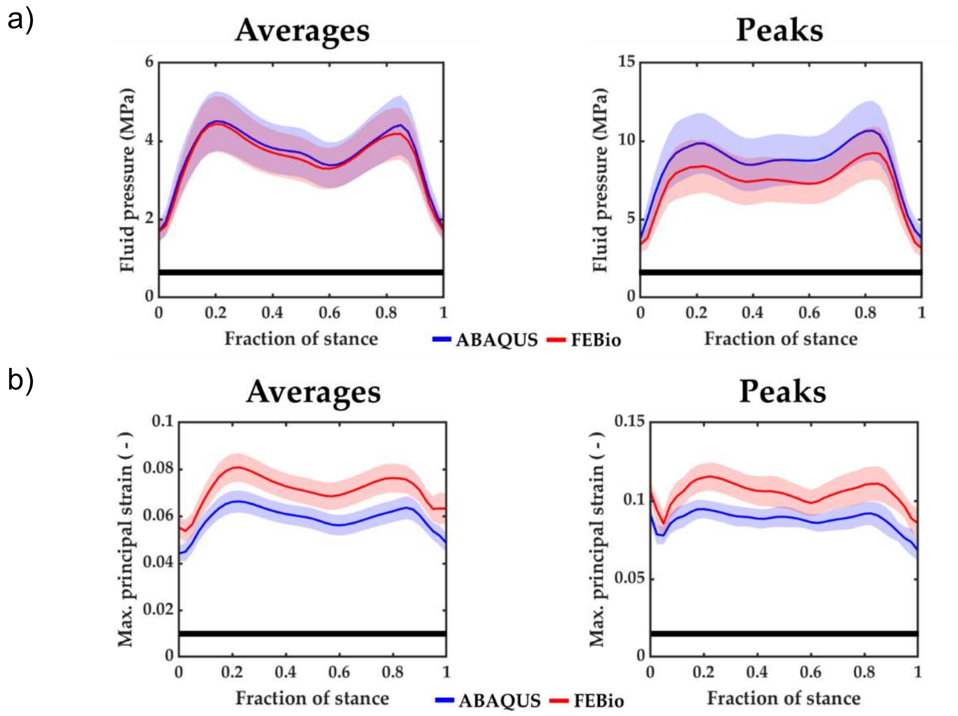

Figure S2. 1D statistical parameter mapping (α=0.05) comparing the average (left) and peak (right) values over the contact area of a) the fluid pressure and b) the maximum principal strain. The black solid line indicates that the two distributions compared differed during the entire loading cycle (p<0.001).

b. Correlations between the differences in results and model parameters

In addition, we compared the mean of the point-wise differences for the stance phase, looking for linear correlations between the results from the different approaches. We defined each point in Fig. S3 as:

$$\overline{X^k} = \frac{1}{N_t}\sum_{i=1}^{Nt}\left(X_i^{FEBio} - X_i^{ABAQUS}\right), \quad (1)$$

$X = \{Fluid\ pressure, Maximum\ principal\ stress, Maximum\ principal\ strain\}$

$\overline{X^k} =\ Mean\ value\ of\ the\ variable\ X\ in\ the\ model\ k$

$N_t = Total\ number\ of\ time\ points\ (i)\ compared$

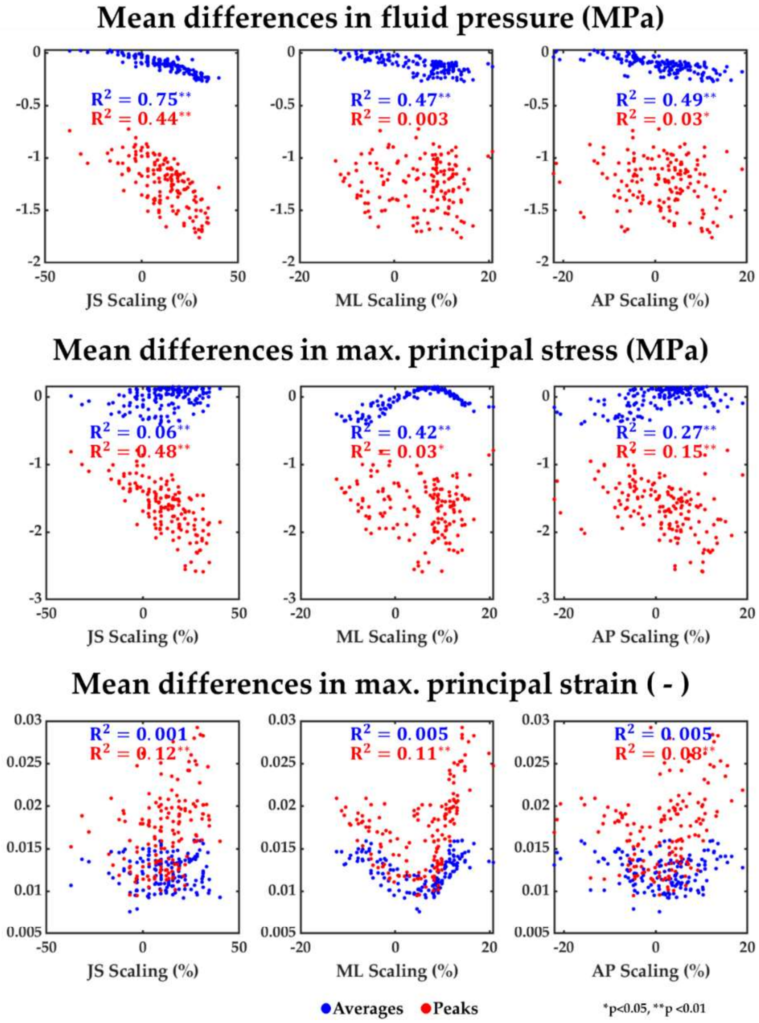

Figure S3. Each point represents the mean difference for one model. Blue for averages and red for maximums over the contact area. The x-axis is the ratio between the dimension in the model with respect to the template $\left(Scaling = \frac{New\ model}{Tamplate\ model} - 1\right)$, for each parameter the measurements of the joint space (JS), medial-lateral (ML), and anterior-posterior (AP).

Fig. 3 suggests an evident correlation between the scaling of the joint space dimension and the differences observed in fluid pressure and maximum principal stress.
